# The Nrf2/HO-1 Axis as Targets for Flavanones: Neuroprotection by Pinocembrin, Naringenin, and Eriodictyol

**DOI:** 10.1155/2019/4724920

**Published:** 2019-11-13

**Authors:** Solomon Habtemariam

**Affiliations:** Herbal Analysis Services UK & Pharmacognosy Research Laboratories, University of Greenwich, Central Avenue, Chatham-Maritime, Kent ME4 4TB, UK

## Abstract

Flavanones are a group of flavonoids that derive from their immediate chalcone precursors through the action of chalcone isomerase enzymes. The Aromatic A and B rings, C4-keto group, and the 15-carbon flavonoid skeleton are all evident in flavanones, but a notable absence of C2-C3 double bond and a lack of oxygenation at C-3 position of the C-ring makes them distinctively different from other groups such as flavonols (e.g., quercetin). On the basis of oxygenation level in the B ring, flavanones can vary from each other as exemplified by pinocembrin (no oxygenation), naringenin (4′-hydroxyl), or eriodictyol (3′,4′-dihydroxyl substitution). These groups are generally weaker free radical scavengers as compared to quercetin and derivatives though eriodictyol has a better free radical scavenging profile within the group due to the presence of the catechol functional moiety. In this communication, their antioxidant potential through the induction of antioxidant defenses is scrutinized. These compounds as exemplified by pinocembrin could induce the nuclear factor erythroid 2-related factor 2- (Nrf2-) heme oxygenase-1 (HO-1) axis leading to amelioration of oxidative stress in cellular and animal models. Their neuroprotective effect through such mechanism is discussed.

## 1. Introduction

Reactive oxygen species (ROS) and/or their free radical derivatives are generated under normal physiological conditions such as the respiratory mitochondrial electron transport pathway or as part of the immune cell defense against pathogenic organisms. While their physiological roles such as signal transduction pathways are well-known, their overproduction or deficiencies in antioxidant defense mechanisms attribute to a host of pathological conditions collectively called oxidative stress (OS). The numerous diseases including neurodegenerative and neuropsychological disorders, diabetes, inflammatory disease, and chronic cardiovascular, pulmonary, and kidney diseases are all good examples of OS-associated diseases. One common understanding in such diseases is that free radicals and/or ROS induce direct damage to macromolecules such as structural proteins and enzymes, DNA, carbohydrates, and lipids [1]. The resulting tissue damage often in combination with exaggerated inflammation leads to cellular apoptosis. The most common ROS are free radicals such as hydroxyl radical (OH^·^), superoxide (O_2_^·–^), and nitric oxide (NO^·^) and nonradical species such as hydrogen peroxide (H_2_O_2_), peroxynitrite (ONOO^−^), and hypochlorous acid. Given that the most active ROS in biological reactions are represented by free radicals such as OH^·^, the inhibition of their formation primarily by limiting the availability of transition metals (e.g., copper and iron ions) or direct radical/ROS scavenging constitutes common mechanisms of antioxidant defenses.

Living organisms are also equipped with a plethora of antioxidant defenses including peptides (glutathione, GSH), dietary antioxidants (vitamins and tocopherols), proteinous metal chelators and transporters, and antioxidant enzymes (e.g., superoxide dismutase (SOD), catalase (CAT), glutathione reductase (GR), and glutathione peroxidase (GPx)). In addition, numerous natural products, mainly polyphenols, from dietary or medicinal plants are employed for their capacity to either remove free radicals through scavenging effect or inhibit ROS generation through chelation with metal ions or boosting antioxidant defenses.

**Figure 1:**
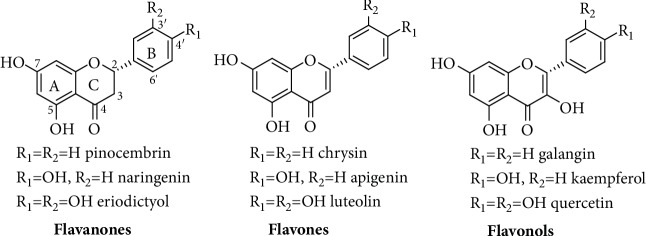
Structures of flavanones and related compounds. Pinocembrin, naringenin, and eriodictyol differ from each other by the degree of oxygenation in the B ring. Notice the difference between flavanones and flavones by the presence of the C2-C3 bond in the latter while flavonols possess further oxygenation at the C-3 position.

With over 8000 compounds isolated from natural sources, flavonoids represent one of by far the most diverse groups of phenolic secondary metabolites. Structurally, they are based on the 15-carbon skeleton constructed in C6-C3-C6 fashion or two phenyl rings (rings A and B) joined together with a three carbon bridge. Flavonoids differ from each other on the basis of B ring attachment position (i.e., C2, C3, or C4 positions), degree of oxygenation and oxygenation patter, presence or absence of the C-ring, presence or absence of C2-C3 double bond, etc. As shown in Figure 1, one noticeable chemical feature of flavanones, which also determines their biological activity such as an antioxidant effect, is the lack of C2-C3 double bond and their stereochemistry at C-2 position. Within the flavanone group, the degree of oxygenation in the B-ring attributes to the variations exhibited by pinocembrin, naringenin, and eriodictyol (Figure 1). Further structural variations could also come through *C-* or *O-*acylation with a range of functional groups (e.g., tiglyl and geranyl) or glycosylation as with other flavonoids.

Undoubtedly, by far the most studied biological activity of flavonoids is related to direct ROS scavenging effect which is a function of their phenolic structure with more prominent activity known to be associated with the diorthohydroxyl (catechol) functional moiety. Hence, numerous structur-activity studies showed the highest radical scavenging effect for flavonols while flavanones, with the exception of eriodictyol, are generally weak [2–4]. Unlike direct ROS scavenging effect, the induction of antioxidant defenses is an attribute of both phenolic and nonphenolic natural products. Hence, many monoterpenoids, ginkgolides, and larger molecular weight terpenes such as ursolic acid and ginsenosides are known to induce antioxidant defenses with implication of therapeutic potential for diverse OS-associated diseases [5–7]. On this ground, the antioxidant potential of the less-potent ROS scavenger flavonoids, flavanones, is scrutinized herein by assessing their ability to induce the known antioxidant defense Nrf2/HO-1 (nuclear factor erythroid 2-related factor- (Nrf2-) heme oxygenase-1 (HO-1)) axis. In view of the known therapeutic potential of natural products for complex diseases such as AD [8], the induction of Nrf2/HO-1 by flavanones as a potential therapeutic strategy for neurodegenerative diseases is also highlighted.

## 2. The Nrf2/HO-1 Axis: Regulation of Antioxidant Genes and Proteins

The nuclear factor erythroid 2-related factor 2 (Nrf2; encoded by the *Nfe2l2* gene) is a transcription factor responsible for the regulation of cellular redox balance in eukaryotic organisms. By regulating the expression of genes that possess the antioxidant/electrophile response elements (ARE/EpRE), it plays a key role in the activities of phase II detoxification enzymes and stress proteins. Among the various well-characterized antioxidant genes/proteins under this regulation are the glutamate-cysteine ligase, glutathione peroxidase 1 (*GPX1*), thioredoxin reductase 1 (Txnrd1), NAD(P)H-quinone oxidoreductase 1 (NQO1), glutathione-*S*-transferase (GST), SOD, CAT, peroxiredoxin (*PRDX1*), ferritin, and heme oxygenase-1 (HMOX1, HO-1). Given this critical cytoprotective regulatory role on genes and proteins, the Nrf2/HO-1 axis in neuroprotection under normal and pathological conditions has recently been the subject of intense research [9–12].

**Figure 2:**
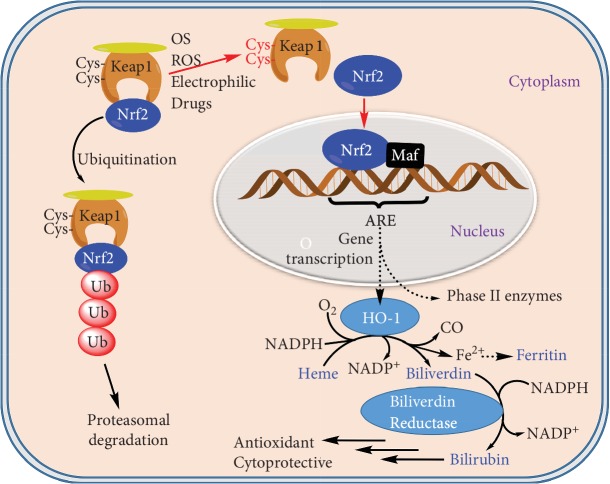
The Nrf2-Keap1-HO-1 pathway. The transcription factor, Nrf2, is sequestered in the cytoplasm by the cysteine- (Cis-) rich Kelch-like ECH-associated protein 1 (Keap1). The binding of Nrf2 with Keap1 is also the basis for its degradation through the ubiquitin- (Ub-) based proteosomal pathway. Under OS or induction by ROS and drugs, the Keap1 response through Cis could lead to the release and stabilization of Nrf2 [27]. The phosphorylation of Nrf2 also leads to its release and translocation into the nucleus. Nrf2 as a conjugate with the Maf proteins binds to the antioxidant response element (ARE) to induce the transcription of target genes including HO-1. The degradation of heme to an antioxidant bilirubin via the biliverdin intermediate is also shown. Other products of the system induce carbon monoxide (CO) and Fe^2+^ which further induce ferritin production.

In the presence of NADPH (nicotinamide adenine dinucleotide phosphate—reduced form), HO-1 catalyzes the rate limiting step in the breakdown of heme to bilirubin [13]. By using oxygen, heme is degraded to liberate Fe^2+^ and carbon monoxide (CO) along with biliverdin which is subsequently acted on by biliverdin reductase to liberate bilirubin (Figure 2). The antioxidant effect of HO-1 is linked to this ability to generate bilirubin which is known to remove ROS including OH, singlet oxygen, and O_2_^·-^ [14–16]. The generation of HO-1 has also been suggested to account to some of the antiapoptotic and anti-inflammatory effects associated with HO-1 induction. Since HO-1 is an inducible enzyme that plays a critical role in the amelioration of OS under various conditions [8–12], its dysregulation or deficiency is associated with a variety of pathologies such as neurodegenerative (AD, Parkinson's disease (PD)), multiple sclerosis, and neuroinflammatory diseases [17–20]. Its significance in diverse OS-related pathologies such as obesity, lung, cardiovascular, and kidney diseases has also been highlighted [21, 22]. Hence, HO-1 knock-out mice are susceptible to neurodegenerative disease development while bilirubin could induce neuroprotective effects both under *in vitro* and *in vivo* conditions [23–26]. Accordingly, the upregulation of HO-1 induction seems to be a reasonable therapeutic approach in OS-related brain diseases.

The basic mechanism through which Nrf2 regulates the expression of hundreds of genes has been extensively researched in recent years. As a transcription factor, Nrf2 is sequestered in the cytoplasm by binding with the Kelch-like ECH-associated protein 1 (Keap1): i.e., Nrf2 exists in the cytoplasm in its inactive state. The Keap1 is a cysteine- (Cys-) rich protein which acts as an OS sensor and interacts with electrophilic compounds, ROS, and others leading to the Nrf2 release/dissociation to move/translocate to the nucleus [27–32]. Keap1 is also known to promote the ubiquitination and degradation of Nrf2, and its inhibition leads to the stabilization and/or increased half-life of Nrf2. Hence, Keap1 responds to OS through oxidation of the cysteine residues leading to its inactivation and subsequent Nrf2 stabilization and/or translocation into the nucleus [33].

By making a complex with small Maf (also called protooncogene c-Maf or V-maf musculoaponeurotic fibrosarcoma oncogene homolog) proteins, Nrf2 in the nucleus binds to the antioxidant responsive elements (ARE) in the promoter region of target genes. Hence, activation of Nrf2 leads to the induction of HO-1 expression along with other antioxidant proteins (see Figure 2) [34].

## 3. Neuroprotective Effects of Flavanones via the Nrf2/HO-1 Axis

The role of the Nrf2/HO-1 axis in neuroprotection by flavanones is mostly studied using the SH-SY5Y cellular model. For pinocembrin (Table 1), pretreatment with up to 25 *μ*M could ameliorate neurotoxicity induced by H_2_O_2_ [35], methylglyoxal [36], paraquat [37], 6-hydroxydopamine (6-OHDA) [38], N-methyl-4-phenyl-1,2,3,6-tetrahydropyridinium (MPP^+^) [39], and amyloid-*β* (A*β*) [40]. Hence, when oxidative stress is induced in neuronal cells either directly via the addition of ROS or through the A*β* model of AD or Parkinson's disease (6-OHDA and MPTP), pinocembrin can induce protective effects. In these models, the antioxidant mechanism of neuroprotection was evident from the induction of the heme oxygenate-1 (HO-1) enzyme and activation of Nrf2. The critical role of the Nrf2-HO-1 axis for the observed neuroprotection was also evident through HO-1 inhibition (with 0.5 *μ*M ZnPP IX) or Nrf2 silencing (Table 1). Moreover, the anti-inflammatory effect of pinocembrin in the lipopolysaccharide- (LPS) stimulated BV-2 microglial cells [41] or H_2_O_2_-treated SH-SY5Y cells [35] through the induction of the Nrf2-HO-1 axis has been shown. Hence, pinocembrin has been shown to inhibit the expression of nuclear factor-*κ*B (NF-*κ*B) and proinflammatory cytokines, inducible nitric oxide synthase (iNOS) and cyclooxygenase-2 (COX-2), as well as prostaglandin-E2 (PGE_2_) production, among others (Table 1). It is also worth noting that pinocembrin can suppress OS through the Nrf2-HO-1 induction in other experimental models such as gentamicin-induced nephrotoxicity in rats or carbon tetrachloride- (CCl_4_-) induced hepatotoxicity and fibrosis in rats [42].

The neuroprotective effect of naringenin (Table 2) through the Nrf2/HO-1 axis is similar with the above-mentioned evidences for pinocembrin. These include SH-SY5Y cells subjected to the toxicity of paraquat [65], H_2_O_2_ [47], 6-OHDA [50]; primary cortical neurons of rats subjected to hypoxia and reoxygenation injury [49] or oxygen and glucose deprivation/reperfusion [46]; or neuron-glia cocultures [43]. *In vivo*, naringenin could also induce protective effect through the Nrf2-OH-1 induction as shown in the aging mice model [45] and the MCAO ischemic stroke model [46].

Readers should also bear in mind that naringenin has been extensively studied for its cytoprotective effect in nonneuronal cells through Nrf2-OH-1 mechanism. This includes inhibition of the CCl_4_-induced liver injury [51, 52]; O_2_^·-^-induced inflammatory pain [53]; tumor necrosis factor-*α*- (TNF-*α*) induced vascular smooth muscle cells (VSMC) proliferation and migration [54]; inflammation and arthritis [55]; acute pancreatitis in mice [56]; ultraviolet light-induced injury in human dermal fibroblasts [57] or ultraviolet B irradiation-induced skin inflammation and oxidative stress in mice [58]; streptozotocin- (STZ) induced pancreatic *β*-cell injury *in vitro* and *in vivo* [59]; glycosylation and NO production in macrophages [60]; protective effect in cardiorenal syndrome [61]; paraquat-induced toxicity in human bronchial epithelial BEAS-2B cells [62]; oxidative stress and cell death in cultured cardiomyoblast [63]; and mucus hypersecretion in human airway epithelial cells [64]. The 7-*O*-glucoside of naringenin has also been shown to protect H9C2 cells from the doxorubicin-induced apoptosis through the Nrf2-OH-1 mechanism [65].

The study on the neuroprotective effects of eriodictyol through the Nrf2/HO-1 pathways is not as diverse as the other flavanones. The limited available data (Table 3) include protection of primary cortical neuron from A*β* [67] and PC12 pheochromocytoma cells from the H_2_O_2_-induced neurotoxicity [69], while the 7-*O*-glucoside of eriodictyol showed protection from brain damage and ameliorated neurological deficits *in vivo* and *in vitro* as evidenced from oxygen and glucose deprivation model in primary astrocytes [68]. More evidences on the role of the Nrf2/HO-1 axis in cytoprotection by eriodictyol came from studies showing protective effect on human retinal pigment epithelial cells from oxidative stress-induced death [70–72], oxidative stress and cell death induced in endothelial cells [73], cisplatin-induced kidney injury [74], and high glucose-induced oxidative stress and inflammation in retinal ganglion cells [75]. Eriodictyol-7-*O*-glucoside has also been shown to induce the Nrf2/HO-1 axis-dependent protection against the cisplatin-induced toxicity [76].

## 4. Neuroprotective Effects of Flavanones through Other Mechanisms

Without a specific mention of the Nrf2/HO-1 axis, numerous studies also showed the neuroprotective effects of flavanones under oxidative stress in neuroinflammatory conditions including cellular (neuronal and glial cultures), stroke, AD, and Parkinson experimental models. Given the role of the Nrf2/HO-1 axis in OS and neurodegenerative conditions (Section 2) as well as the specific effect of flavanones in this system (Section 3), the wider neuroprotective effect of these compounds are further outlined below.

### 4.1. Pinocembrin

A number of studies have been devoted to assessing the neuroprotective effect of pinocembrin by using the cerebral ischemia/reperfusion (I/R) injury in animal models. In these studies, the compound was shown to be effective at doses as low as 5 mg/kg in rats when administered (i.v.) 30 min before ischemia [77]. At doses between 2.5 and 10 mg/kg (i.v.), pinocembrin can also ameliorate neurological deficits in mice induced by intracerebral hemorrhage (ICH) [70]. This effect was also coupled with anti-inflammatory mechanism as microglial activation along with the expression of proinflammatory cytokines (TNF-*α*, interleukin- (IL-) 1*β* and IL-6, and toll-like receptor 4 (TLR4)) was downregulated: an effect which was also observed in the LPS-stimulated BV-2 cells or primary microglial cells (decreased M1-related cytokines and markers (IL-1*β*, IL-6, TNF-*α*, and iNOS), NF-*κ*B activation, and TLR4 expression) [78]. Pinocembrin could also reduce the number of classically activated M1-like microglia without affecting M2-like microglia in the perilesional region. Pinocembrin has further been shown to be effective in the rat thromboembolic stroke model (10 mg/kg, intravenous (i.v.)) or *in vitro* at just 1 *μ*M concentration when the human cerebral microvascular endothelial cells were used as a blood-brain barrier (BBB) model [79]. In an oral therapy regimen, pretreatment with pinocembrin at 10 mg/kg (daily for 7 days) could also ameliorate the global cerebral ischemia-reperfusion injury in rats [80] or when administered (5 mg/kg, i.v.) at 30 min before ischemia and 30 min, 2 h, or 6 h after reperfusion [81, 82]. Other studies also showed similar outcomes for the same dose through i.v. administration during the reperfusion phase [83, 84]. Administration of pinocembrin immediately after reperfusion (1, 5, and 10 mg/kg, i.v.) [84] or after 16 h after the middle cerebral artery occlusion (MACO) [85] or up to 23 h in the MCAO model [87] could also be neuroprotective. Common carotid artery ligation as a model of chronic cerebral hypoperfusion was also employed to demonstrate cognitive enhancement and neuroprotective effect by pinocembrin (0.5 mg/kg; 5.0 mg/kg, i.p. for 13 days) [88].

In the *in vitro* model of AD where SH-SY5Y cells were subjected to the toxic effect of A*β*_25-35_, pinocembrin (1-20 *μ*M) has been shown to ameliorate apoptosis and/or mitochondrial dysfunctions including reversal of decreased membrane potential and Bcl-2/Bax ratio [89]. In A*β* AD model using APP/PS1 transgenic mice, pinocembrin treatment (40 mg/kg p.o. for 12 weeks) could prevent the cognition decline without altering A*β* burden and oxidative stress [89]. On the other hand, newborn mouse neuronal parenchymal cultures subjected to fibrillar A*β*_1–42_ could be protected by pinocembrin. This neuroprotection in both *in vitro* and *in vivo* models was accompanied by inhibition of glial activation, receptor for advanced glycation end products- (RAGE-) induced p38 mitogen-activated protein kinase (MAPK) pathway, and inflammatory mediators [90]. It is worth noting that the inhibition of upregulation of RAGE transcripts and protein expression both *in vivo* and *in vitro* by pinocembrin was not extended to the suppression of A*β*_1-42_ production and scavenging intracellular ROS [91]. The memory enhancement by pinocembrin in these models is thus not associated with a direct ROS scavenging effect. The effect of pinocembrin in neuronal cells was also evident in human brain microvascular endothelial cells as cytoprotection (at 3-30 *μ*M) from fibrillar A*β*_1-40_ was demonstrated [90].

The MPP^+^-induced SH-SY5Y neurotoxicity as a valid model of PD could also be ameliorated *in vitro* by pinocembrin at 1-20 *μ*M range [39, 92]. When these cells were also subjected to 6-OHDA-induced cell death, pinocembrin could be equally protective [38]. Other neuroprotective models studied for pinocembrin include the glutamate-induced apoptosis in SH-SY5Y cells where doses from 0.1 to 10 *μ*M have been shown to be effective [93]. Primary cortical neuronal cultures subjected to oxygen-glucose deprivation/reoxygenation also responded well to pinocembrin treatment (0.1, 1, and 10 *μ*M) as with the ischemia/reperfusion-like injury—transient focal cerebral ischemia model in rats (3, 10, and 30 mg/kg, i.v.) [94].

### 4.2. Naringenin

PubMed (https://www.ncbi.nlm.nih.gov/pubmed) search on naringenin for inflammation and oxidative stress resulted in 142 and 192 hits, respectively. Through such general mechanisms and several specific actions against enzymes and receptors, naringenin can induce cytoprotective effect in a number of cell/tissue systems. One of the most researched pharmacological effect of naringenin is AD where it showed protective effect against A*β*-induced toxicity both in cellular (e.g., SH-SY5Y cells) and animal models [95, 96]. This includes increasing the levels of A*β* degradation enzymes in M2 microglial cells [97] or by inhibiting the release of NO, the expression of iNOS, and COX-2, as well as proinflammatory cytokines in microglia [98]. Furthermore, this anti-inflammatory effect of naringenin was shown to be correlated with the induction of suppressors of cytokine signaling- (SOCS-) 3 expression in microglia. In SH-SY5Y cells, naringenin downregulates the amyloid precursor protein (APP) and *β*-site amyloid precursor protein cleaving enzyme (BACE) expression [95], while in PC12 cells the cytoprotective effect of naringenin against A*β*_25-35_ was shown to be mediated via promoting protein kinase B (Akt) and glycogen synthase kinase-3*β* (GSK-3*β*) activation and inhibition of cell apoptosis and caspase-3 activity [99]. In the rat model of iron-induced neurotoxicity in the cerebral cortex, naringenin ameliorates neurotoxicity by increasing antioxidant defenses (dismutase and catalase and in the levels of nonenzymatic antioxidants like total thiols and ascorbic acid) [100]. Other favorable effects of naringenin include the STZ-induced dementia model of rats, where in addition to behavioral markers, reversal of tau hyperphosphorylation in both the hippocampus and cerebral cortex was observed via inhibition of GSK-3*β* activity [101]. The level of A*β* in the brain was also shown to be reduced by naringenin treatment [101]. This is consistent with other studies that showed reversal of impaired learning and memory ability in the STZ-induced AD model in rats [102–104]. In addition to neuroprotective effect in the cellular system (PC12 cells) from A*β*-induced toxicity, naringenin has been shown to ameliorate the scopolamine-induced amnesia in mice [105, 106].

In silico docking and *in vitro* enzyme inhibition studies have shown that naringenin was among the citrus flavonoids that showed BACE1 and cholinesterase inhibitory potential [107]. Other studies however also confirmed these results as a weak activity [108].

**Figure 3:**
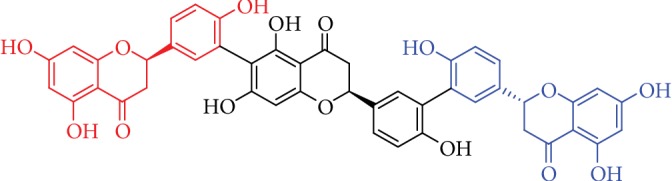
Structure of selagintriflavonoid A. The three units of naringenin are shown in different colours.

A similar neuroprotective profile has also been demonstrated for naringenin in PD models. In primary rat neuronal and glial cultures, neurotrophic effects to support dopaminergic neuron survival and elicitation of astrogliosis and neurotrophic factor release have been demonstrated [43]. In a classical PD model of the 1-methyl-4-phenyl-1,2,3,6-tetrahydropyridine- (MPTP-) induced neurotoxicity in mice, naringenin has been shown to reverse the toxic effects of MPTP as revealed by the lower level of lipid peroxidation (LPO) and increased activities of antioxidant enzymes (GR and CAT) along with improved behavioral performance [109]. This study also revealed suppression of the MPTP-induced iNOS expression in the mice brain in general and neuronal viability in the substantia nigra and striatal regions of MPTP-intoxicated mice. In this model, the MPTP-induced *α*-synuclein was downregulated while enhancing the dopamine transporter and tyrosine hydroxylase (TH) protein expressions [110]. As expected, naringenin also downregulated TNF-*α* and IL-1*β* mRNA expressions and improved SOD levels [110]. The 6-OHDA-induced neurotoxicity in SH-SY5Y cells could also be ameliorated by naringenin [111]. This data was consistent with *in vivo* studies using the 6-OHDA model of PD in rats where the number of TH-positive cells in the substantia nigra and dopamine levels in the striata were enhanced by naringenin treatment [50]. Other relevant neuroprotective effects by naringenin were in the rotenone-induced PD rat model [112] or primary mesencephalic cultures subjected to oxidative damage by H_2_O_2_, 4-hydroxynonenal, rotenone, 6-OHDA, or MPTP [113]. On the other hand, selagintriflavonoid A, a compound that bears three naringenin units in its structure (Figure 3), has been shown to display BACE1 inhibition with IC_50_ value of 0.75 ± 0.05 *μ*M [114]. By binding and inhibiting the phosphorylation of collapsin response mediator protein-2 (CRMP-2), naringenin has been shown to exert possible therapeutic potential for AD [115]. From studies such as hyperphosphorylated protein levels in AD (e.g., collapsin response mediator protein (CRMP-2)), the effect of naringenin-7-*O*-glucuronide was further shown to be similar with naringenin [116].

### 4.3. Eriodictyol

Among the flavanones, eriodictyol is the least studied for its potential neuroprotective effects. This is despite the fact that eriodictyol is a far better antioxidant as direct scavenger of ROS than pinocembrin and naringenin. In the LPS-induced biochemical and cognitive impairment studies in mice, eriodictyol (100 mg/kg) has been shown to improve memory along with suppression of amyloid (A*β*_1-42_) burden while the neurotransmitter imbalance (acetylcholine (ACh) level and choline acetyl transferase (ChAT) activity) was restored [116]. The other parameters worth mentioning were suppression of the LPS-induced glial overactivation and regulation of inflammatory cytokine expression via inhibition of the NF-*κ*B and MAPK pathways [116]. By using neuronal damage, motor and memory deficits induced by permanent middle cerebral artery occlusion (pMCAO) model in mice, eriodictyol has been shown to abolish neuronal death, reduce infarct area, and improve neurological and memory deficits induced by brain ischemia [117]. The increased myeloperoxidase (MPO) activity and expression of TNF-*α*, iNOS, and glial fibrillary acidic protein (GFAP) during ischemia could also be abrogated by eriodictyol [117]. In primary cultured neurons subjected to A*β*_25-35_-induced oxidative cell death, eriodictyol (20, 40, and 80 *μ*M) has been shown to ameliorate apoptosis [118]. In the study using BV-2 microglial cells and mouse brain, eriodictyol could alleviate the LPS-induced oxidative stress, cell viability, and mitochondrial potential [68]. Eriodictyol-7-*O*-glucoside could also protect against cerebral ischemic injury in animals and primary cultured astrocytes *in vitro* from oxygen and glucose deprivation- (OGD-) induced oxidative insult [119].

**Figure 4:**
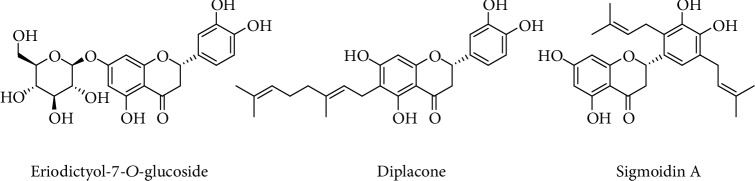
Examples of bioactive eriodictyol derivatives.

A comparative activity screening indicated that a geranyl group at C6 position of eriodictyol is crucial for both human acetylcholinesterase (hAChE) and butyrylcholinesterase (BchE) inhibition. For example, diplacone (Figure 4) showed a 250-fold higher efficacy than its parent eriodictyol. The reported IC_50_ of diplacone was 7.2 *μ*M for hAChE and 1.4 *μ*M for BchE [119]. The addition of prenyl groups in eriodictyol (e.g., sigmoidin A, Figure 4) has also been shown to enhance biological activity in the cellular system even though a similar direct free radical scavenging effect was shown in a cell-free system [120]. In enzyme inhibition assays, such as pancreatic lipase, sigmoidin A was also about 30 times more potent than eriodictyol [121]. Hence, the lipophilic prenyl/geranyl addition in the A or B rings appears to modify the biological activity of eriodictyol. Similar enhancement of biological effects for prenyl derivatives of naringenin has also been reported [122].

## 5. General Summary and Conclusion

Flavonoids are among the most diverse and ubiquitous natural products that are commonly found in fruits and vegetables, food ingredients, and medicinal plants. Among the best studied pharmacology of flavonoids is their antioxidant effect which is linked to their phenolic nature that contributes to metal chelation and direct free radical/ROS scavenging effects. Due to their multifunctional nature which also includes enzyme inhibition and modulation of numerous receptors, the therapeutic potential of flavonoids in complex diseases is often advocated [123]. One further emerging role as antioxidant therapy in recent years is induction of the Nrf2/HO-1 axis which is an attribute of both phenolic and nonphenolic compounds. In this regard, the flavanones represented by pinocembrin are the best examples of flavonoids that lack the catechol functional moiety, the C2/C3 double bond, and C-3 hydroxylation which would enhance metal chelation capacity. Interestingly, the study on pinocembrin as an inducer of the Nrf2/HO-1 axis is more diverse than the better free radical scavenger, eriodictyol (Tables 1 and 3). Although direct comparison of these compounds under the same experimental condition would have been helpful, hence calling for more research in this area, the available data suggest that induction of Nrf2/HO-1 by flavanones is not an attribute of direct ROS scavenging effect, i.e., flavanones are generally weaker antioxidants than related compounds such as flavonols [2–4]. Hence, numerous compounds with weak direct free radical scavenging effects including nonphenolic compounds can also induce neuroprotective effects through induction of Nrf2/HO-1 both *in vitro* and *in vivo* [e.g., 2–6].

As shown in Tables 1–3, the expression of HO-1 is not just a mere coincidence of flavanone action in neuronal cells as abrogating the expression of Nrf2/HO-1 could also ameliorate the observed neuroprotective effects. Hence, the Nrf2/HO-1 pathway is critical for the neuroprotective effect of flavanones. The evidences presented herein also include both *in vitro* and *in vivo* models of AD, PD, brain ischemia models, etc. One can thus see the broad range of therapeutic potential for this group of flavonoids in neurodegenerative diseases. Another interesting research area of development for the Nrf2/HO-1 axis is in neural regeneration. For example, overexpression of HO-1 in the substantia nigra has been shown to enhance the expression of the glial cell-derived neurotrophic factor (GDNF) [124–126]. The association of HO-1 with neurotrophic factors in AD, ischemia, and stroke models has also been established [127–129]. Given the emerging evidences of neurotrophic induction by natural products under neurodegenerative diseases and traumatic brain injury conditions [130], the link between flavanones' effect on Nrf2/HO-1 and neuroregeneration potential adds further therapeutic scopes.

The exact mechanism of induction of Nrf2/HO-1 by the flavanones remains to be elucidated. Wang et al. [49] have shown the critical role of the PI3K/Akt signaling pathway in the induction of the Nrf2/HO-1 axis by naringenin and its protective effect against the D-galactose-induced memory loss in aging mice. The PI3K signaling was also shown to be critical for the anti-inflammatory effect of pinocembrin in microglial cells [40], while the ERK1/2 pathway was shown to be pivotal for the neuroprotective effect of pinocembrin in SH-SY5Y cells [35, 36, 38]. The crucial role of the MAPK and PI3K signaling pathways in the antioxidant gene activation via Nrf2 induction has already been established, and compounds like daphnetin, lonchocarpine, and alfalfa flavonoids exert a cytoprotective effect through such mechanisms [131–133].

Optimizing the neuroprotective effects of flavanones requires more research in the various medicinal chemistry and pharmacokinetic fields. Few studies on the 7-*O*-glucoside derivatives of flavanones are highlighted in this paper. While glycosylation increases water solubility and hence a better bioavailability profile, some studies on the prenyl and geranyl derivatives have been highlighted with better enzyme inhibitory and bioactivity profiles. Another interesting observation is the polymerization of flavanones as evidenced by the favorable biological activity profile of selagintriflavonoid A [114]. Whether this *in vitro* effect would be reproduced in animal models, however, remains to be established. In the meantime, flavanones as evidenced by pinocembrin, naringenin, and eriodictyol appear to have neuroprotective effects through induction of the Nrf2/HO-1 axis. More studies on the optimization of biological activity of these compounds are thus well-merited.

## Figures and Tables

**Table 1:** Neuroprotective effect of flavanones through the Nrf2/HO-1 mechanism: pinocembrin.

Compound	Experimental model	Key findings	Reference
Pinocembrin	Human neuroblastoma SH-SY5Y cells exposed to hydrogen peroxide (H_2_O_2_) (4 h pretreatment (25 *μ*M))	Protects cells from H_2_O_2_-induced cell death and mitochondrial depolarization; ameliorates redox impairment in mitochondrial membranes; suppresses O_2_- and NO production; recovers the suppressed tricarboxylic acid (TCA) cycle enzymes aconitase, alpha-ketoglutarate dehydrogenase, and succinate dehydrogenase; inhibits the activation of NF-*κ*B and levels of IL-1*β* and TNF-*α*; all effects were dependent on the HO-1 enzyme and on the activation of Nrf2—confirmed by HO-1 inhibition (with 0.5 *μ*M ZnPP IX) or Nrf2 silencing (with small interfering RNA (siRNA)).	de Oliveira et al. [35]

Pinocembrin	SH-SY5Y cells exposed to methylglyoxal (pretreatment with 0-25 *μ*M for 4 h)	Ameliorates mitochondrial dysfunction—decreases lipid peroxidation, protein carbonylation, and protein nitration in mitochondrial membranes; suppresses mitochondrial free radical production; increases glutathione (GSH) level in mitochondria; rescues mitochondrial membrane potential (MMP); inhibits cell death through activation of the extracellular-related kinase (Erk1/2) and consequent upregulation of Nrf2; increases the levels of GPx, GR, HO-1, and mitochondrial GSH; all effects could be abolished by silencing of Nrf2 with siRNA.	de Oliveira et al. [36]

Pinocembrin	SH-SY5Y neuroblastoma cells exposed to paraquat (pretreatment for 4 h)	Suppresses the levels of Bcl-2-associated X protein (Bax); inhibits cytochrome c release to the cytosol and caspase-9 and caspase-3 activation; inhibits mitochondrial dysfunction by ameliorating the inhibition of complexes I and V; inhibits the loss of MMP and the decline in ATP levels; antioxidant effects on mitochondria coupled with decreased levels of redox impairment markers; enhances the levels of mitochondrial GSH; effect dependent on activation of the Erk1/2-Nrf2 axis—inhibition of Erk1/2 or silencing of Nrf2 abrogated these effects.	de Oliveira et al. [37]

Pinocembrin	SH-SY5Y cells exposed to neurotoxin 6-hydroxydopamine- (6-OHDA-) induced cell death in pretreatment for 4 h	Improves cell viability and apoptotic rate and decreases Bcl-2/Bax ratio; inhibits oxidative stress (ROS, the level of malondialdehyde, MMP, and SOD); increases Nrf2 protein levels and subsequent activation of ARE pathway genes of HO-1 and gamma-glutamylcysteine synthetase (*γ*-GCS); treatment with Nrf2 small interfering RNA abolished protective effects.	Jin et al. [38]

Pinocembrin	SH-SY5Y exposed to neurotoxic 1-methyl-4-phenylpyridinium (MPP^+^)	Inhibits the induced cell death by increasing HO-1 expression—inhibitor of HO-1 zinc protoporphyrin IX abolished the neuroprotective effect; induces phosphorylation of ERK1/2—cytoprotective effect can be abolished by ERK1/2 inhibitors.	Wang et al. [39]

Pinocembrin	SH-SY5Y cells exposed to A*β*_25-35_ (4 h pretreatment)	Inhibits mitochondrial dysfunctions (lowered MMP, decreased Bcl-2/Bax ratio); inhibits cytochrome c release and caspase-3 cleavage; increases protein levels of Nrf2 and induces HO-1 expression; neuroprotective effects abolished by RNA interference-mediated knockdown of Nrf2 expression or HO-1 inhibitor zinc protoporphyrin IX (ZnPP).	Wang et al. [40]

Pinocembrin	LPS-stimulated BV-2 microglial cells	Inhibits TNF-*α*, IL-1*β*, NO, and PGE_2_ production; suppresses iNOS and COX-2 expression; inhibits phosphoinositide 3-kinase (PI3K), Akt phosphorylation, and NF-*κ*B activation; induces nuclear translocation of Nrf2 and expression of HO-1.	Zhou et al. [41]

**Table 2:** Neuroprotective effect of flavanones through the Nrf2/HO-1 mechanism: naringenin.

Compound	Experimental model	Key findings	Reference
Naringenin	Primary rat midbrain neuron-glia cocultures	Shows concentration- and time-dependent neurotrophic effects to support dopaminergic neuron survival—effect dependent on astroglia; elicits astrogliosis and neurotrophic factor release; increases Nrf2 mRNA and protein expressions both in neuron-glia and astroglia-enriched cultures—Nrf2-siRNA inhibited the induced astrogliosis and neurotrophic factor release; or astroglial Nrf2-siRNA abolished the induced neurotrophic effects on neurons.	Wang et al. [43]

Naringenin	SH-SY5Y cells exposed to paraquat (pretreatment with 80 *μ*M for 2 h)	Decreases the levels of proinflammatory cytokines (IL-1*β* and TNF-*α*) and production of NO; downregulates the levels of COX-2 and iNOS and the activation of NF-*κ*B—the induced antiapoptotic and anti-inflammatory effects were abolished by ZnPP IX (a specific inhibitor of HO-1) or by knockdown of Nrf2 by small interfering RNA (siRNA).	de Oliveira et al. [47]

Naringenin	Lipopolysaccharide- (LPS-) induced cognitive decline in rat (25, 50, or 100 mg/kg/day p.o. for one week)	Dose-dependent improvement in memory and learning; lowers hippocampal malondialdehyde (MDA); improves antioxidant defensive system (SOD, CAT, and GSH); decreases acetylcholinesterase (AChE) activity; lowers hippocampal NF-*κ*B, TLR4, TNF-*α*, COX-2, iNOS, and glial fibrillary acidic protein (GFAP) level and its immunoreactivity; elevates Nrf2.	Khajevand-Khazaei et al. [44]

Naringenin	SH-SY5Y cells exposed to H_2_O_2_ (pretreatment with 80 *μ*M for 2 h)	Reduces LPO, protein carbonylation, and protein nitration in mitochondrial membranes; prevents the functional impairment of the enzymes aconitase, alpha-ketoglutarate dehydrogenase, and succinate dehydrogenase; restores the activities of the complexes I and V; suppresses the induced mitochondria-related apoptosis; promotes an increase in the levels of both total and mitochondrial GSH—silencing of Nrf2 abolished the protective effects.	de Oliveira et al. [65]

Naringenin	*In vitro*, cortical neuron cells isolated from the brains of neonatal rats subjected to oxygen and glucose deprivation/reperfusion or middle cerebral artery occlusion (MCAO ischemic stroke model)	Promotes cortical neuron cell proliferation and inhibits apoptosis and OS; regulates the localization of Nrf2 protein alleviated cerebral oedema; improves neurological defects and reduces apoptosis and OS—silencing Nrf2 mitigate the protective effect.	Wang et al. [46]

Naringenin	Neurons isolated from the brains of rats (model group received hypoxia and reoxygenation treatment, with 20, 40, and 80 *μ*M treatment)	Reduces (80 *μ*M) the levels of ROS; improves mitochondrial dysfunction (increased levels of high-energy phosphates, enhanced mitochondrial transport activity, and increased MMP); increases cell viability and decreases the rate of cell apoptosis; increases the expression of Nrf2 and its downstream target genes.	Wang et al. [49]

Naringenin	Aging mice exposed to D-galactose (open field test and Morris water maze test)	Activates PI3K/Akt signaling and promotes the nuclear translocation of Nrf2 and induces the expression of HO-1 and NAD(P)H-quinone oxidoreductase 1; enhances antioxidant defenses (SOD, CAT, and thiobarbituric acid reactive substance (TBARS) assays).	Zhang et al. [45]

Naringenin	6-Hydroxydopamine- (6-OHDA-) induced neurotoxicity in models of PD both *in vitro* (SH-SY5Y cells) and *in vivo* (mice)	Increases the Nrf2 protein levels and subsequent activation of ARE pathway genes *in vitro* and *in vivo*; protects neuronal cells from oxidative insults—effect dependent on Nrf2 (Nrf2 siRNA abolished the neurotoxic or induction of Nrf2-dependent cytoprotective genes); preserves nigrostriatal dopaminergic neurodegeneration and ameliorates oxidative damage *in vivo*.	Lou et al. [50]

**Table 3:** Neuroprotective effect of flavanones through the Nrf2/HO-1 mechanism: eriodictyol.

Compound	Experimental model	Key findings	Reference
Eriodictyol	A*β*_25-35_ peptide-induced oxidative cell death in primary cortical neurons (20, 40, and 80 *μ*M)	Attenuates the induced apoptosis and activation of c-Jun N-terminal kinases (JNK)/p38 signaling pathway; increases Nrf2 protein levels and subsequent activation of ARE pathway genes in primary cultured neurons—protective effects attenuated by RNA interference-mediated knockdown of Nrf2 expression.	Jing et al. [67]

Eriodictyol-7-*O*-glucoside	Cultured primary astrocytes—oxygen and glucose deprivation-induced oxidative insult; rat model of focal cerebral ischemia	Protects against the induced cell death; increases the nuclear localization of Nrf2 and induces the expression of the Nrf2/ARE-dependent genes—protective effect abolished by RNA interference-mediated knockdown of Nrf2 expression; reduces the amount of brain damage and ameliorates neurological deficits *in vivo*.	Jing et al. [68]

Eriodictyol	Cultured rat pheochromocytoma (PC12) cells exposed to H_2_O_2_-induced neurotoxicity (20, 40, and 80 *μ*M)	Inhibits apoptosis; induces the nuclear translocation of Nrf2, enhances the expression of HO-1 and *γ*-GCS, and increases the levels of intracellular GSH—Nrf2 small interference RNA abolished the induced HO-1 and *γ*-GCS expression and cytoprotective protective effects.	Lou et al. [69]
